# Biotin Interference in Point of Care HIV Immunoassay

**DOI:** 10.1089/biores.2020.0038

**Published:** 2020-11-24

**Authors:** Mohan Kumar Haleyur Giri Setty, Sherwin Lee, Julia Lathrop, Indira K. Hewlett

**Affiliations:** ^1^ Laboratory of Molecular Virology, Office of Blood Research and Review, CBER, FDA, Silver Spring, Maryland, USA.; ^2^ Office of Blood Research and Review, CBER, FDA, Silver Spring, Maryland, USA.

**Keywords:** HIV, biotin, HIV-1 p24, seroconversion panel, antigen, antibody

## Abstract

The use of high concentrations of biotin as a dietary supplement to improve hair, skin, and nail quality has increased in the United States over the past few years. High concentrations of biotin have been shown to interfere with some diagnostic assays that use streptavidin–biotin interactions as one of the steps in the assay. The objective of this report is to evaluate potential biotin interference on the analytical and clinical sensitivity of a point of care (POC) antigen–antibody combo HIV-1 assay. We spiked biotin at concentrations ranging from 12.5 to 400 ng/mL into serum and plasma containing HIV-1 subtype B p24 antigen derived from culture supernatant. The p24 antigen was present in the matrices at 30 pg/mL. Fifty microliters of each sample was applied to Alere Determine HIV-1/2 Ag/Ab combo assay strips in duplicate and results were read by eye after 20 to 30 min. Biotin interfered with detection of HIV-1 p24 in serum and plasma. HIV-1 p24 was not detected at 30 pg/mL p24 when biotin was present at 200 ng/mL concentration. Our study demonstrated that elevated levels of biotin in samples may interfere with POC assays. It is important to consider biotin supplements as potential sources of falsely increased or decreased test results, especially in cases wherein supplementation cannot be ruled out.

## Introduction

Biotin–streptavidin interactions are the strongest known noncovalent biological interactions, with a femtomolar range dissociation constant and an interaction that is resilient to temperature and pH changes.^1^ Biotin is used together with streptavidin in the design of many diagnostic assays, leveraging the high stability and specificity of the interactions to increase analytical sensitivity.^2^ In some designs, immobilized streptavidin (e.g., on a bead or membrane) is used to capture a biotin-labeled analyte. The stable biotin label on an analyte is small and rarely interferes with the function of labeled molecules, enabling the streptavidin–biotin interaction to be used for development of robust and highly sensitive assays.

Assays that use biotin–streptavidin chemistry to capture and evaluate an analyte may be susceptible to interference from free excess biotin in a sample if it competes with the biotin-labeled analyte for binding to streptavidin. Normal intake of biotin at the daily recommended dietary allowance of 30 μg/day (123 nmol/day) from food rarely interferes with assay results.^3^

The publication of several case studies that biotin has beneficial effects on multiple sclerosis has increased prescription use of high dose of biotin for these patients, and with many other inherited disorders causing an increase in the number of people taking biotin orally.^4^ Also, in recent years there has been an increase in dietary biotin supplementation by the general public, ostensibly to improve hair and nail growth, with a concurrent increase in reports of biotin interference in laboratory tests.^5^ Limited studies have demonstrated that oral intake of 1 to 100 mg biotin results in mean peak serum biotin concentrations of 8.6 and 495 ng/mL 1 and 3 h after ingestion, with peak levels declining with half-lives of 8 to 19 h.^6^ Studies have shown that no biotin interference was observed in some laboratory tests in samples from healthy individuals whose biotin concentrations ranged from 0.12 to 0.36 nM in blood and this concentration range was tolerated by most assay formats and has negligible interfering effects on streptavidin–biotin interaction.^7,8^ The FDA has issued two safety communications detailing concerns regarding biotin interference with laboratory tests. Therefore, it is necessary to evaluate assays that use biotin–streptavidin in their design to determine the levels of biotin that interfere (if any) with each assay.^9^

Biotin interference in immunoassays varies depending on the format of the assay and can result either in falsely high or low falsely analyte detection depending on the design.^10,11^ Certain types of biotin–streptavidin assays are more susceptible to inhibition; the most vulnerable are those assays wherein the biotinylated capture reagent is bound to the solid phase. In sandwich assays, excess biotin can compete with biotinylated antibodies, artificially decreasing the apparent analyte concentration. In competitive assays, excess biotin competes with the biotinylated analogue for streptavidin binding sites, artificially increasing the apparent analyte concentration.

Recent reports have shown errors in laboratory testing assays due to biotin–streptavidin interactions for prostate cancer, pancreatic function, ovarian cancer, pituitary function, vitamin deficiency, tumor marker, and thyroid function panels.^11^ An outpatient survey of biotin supplement usage showed that 7.7% of individuals attending the clinic used biotin supplementation.^4^ The studies and case reports already noted involved testing performed on automated laboratory systems for general human analytes. There is little information regarding biotin interference in point of care (POC) HIV immunoassays, therefore, we conducted a small study using a rapid POC HIV-1 antigen–antibody combo assay, which are important for HIV-1 diagnosis.

We focused on HIV-1 p24 in our study, which is the antigen component in antibody–antigen assays, also called combination, or combo assays. Viral capsid HIV-p24 Gag protein is a structural protein with highly conserved amino acid sequences present in a large quantity early in infection compared with other viral proteins, making it a useful marker for early diagnosis of HIV-1 infection.^12^ Therefore, inclusion of HIV-1 p24 in tests can reduce the diagnostic window period by 4–5 days compared with that of antibody-only assays.^13,14^

## Materials and Methods

We purchased base matrix plasma from a Seracare (processed human plasma after removal of fibrin and lipids), to determine the concentration range of HIV-1 p24 and biotin to be used in the studies. Clinical seroconversion panels were purchased from Seracare; the seroconversion panels included panel members that are positive for antigen only, antibody only, and for both antigen and antibody (Table 2). We used phosphate-buffered saline (PBS) from Quality Biologics, Biotin and Triton X-100 from Sigma Aldrich. The HIV Ag/Ab test used was the Alere Determine™ HIV-1/2 Ag/Ab combo test from Abbott Laboratories, which is the only FDA-approved POC Ag/Ab test. Stocks of HIV-1 p24 culture supernatant were from stocks previously grown in our laboratory in peripheral blood mononuclear cells and aliquoted for use. After initial experiments with multiple matrices described hereunder, we chose 30 pg/mL of HIV-1 p24 culture supernatant in base matrix plasma and serum for our study. We spiked 30 and 60 pg/mL of HIV-1 p24 culture supernatant and 25 ng/ml to 800 ng/mL of biotin to obtain final concentrations of HIV-1 p24 of 15 and 30 pg/mL in base matrix plasma and serum. Biotin concentrations of 12.5, 25, 50, 100, 200, and 400 ng/mL were prepared in serum and plasma.

**Table 2.** Alere Determine HIV Ag/Ab Combo Assay Seroconversion Plasma

Sample ID	Antibody	Antigen
PBR-975-05	Negative	Positive
PBR946-03	Negative	Positive
PBR946-04	Negative	Positive
PBR947-02	Positive^a^	Positive
PBR947-03	Positive	Negative^b^
PBR947-04	Positive	Negative

^a^ Very faint on Alere.

^b^ Alere did not detect.

### HIV-1 p24 antigen testing strategy

An initial study was performed to determine the concentration of HIV-1 p24 spiked into plasma that produced reactive results in the assay. In our initial study, HIV-1 p24 culture supernatant was lysed with 0.1% of Triton-X-100 by vortexing gently before use. Different 1 × concentrations of culture supernatant-derived p24 were prepared (1, 5, 10, 15, 20, 25, 50, 100 pg/mL) in PBS, serum, and plasma. In our main study, biotin stock at 1 mg/mL was prepared in 0.01 M NaOH and further diluted in distilled water to a concentration of 800 ng/μL. One microliter of this stock was added to 1 mL of each matrix to produce a final concentration of 800 ng/mL of biotin. Biotin and HIV-1 p24 stocks of 2 × final concentration were prepared separately and mixed in equal volume to obtain the desired 1 × concentrations. We decided to use 30 pg/mL of HIV-1 p24 in plasma and serum for our final biotin interference studies based on our initial results.

For all our experiments with the Alere Determine HIV-1/2 Ag/Ab combo assay, the test was performed following the manufacturer's instructions. Test strips were placed on the workstation provided, and the covers of the strips were removed to apply 50 μL of the plasma, serum, or PBS spiked with the HIV-1 p24/biotin solutions to the sample pad. Appropriate controls for p24 and biotin were also used in the study. The results were evaluated by eye for the presence or absence of a band at the antigen detection site of the strip 20 min after sample loading. Similarly, we tested seroconversion samples by directly spiking 10, 20, and 40 ng of biotin in 5 to 100 μL of each seroconversion panel member and tested 50 μL on the Alere strips, and all tests were done in duplicates.

## Results and Discussion

From our study using culture supernatant-derived HIV-1 p24 spiked into plasma or serum, 15 pg/mL produced a very faint band and 30 pg/mL produced a strong band indicating reactivity on the Alere Determine HIV-1/2 Ag/Ab combo test. Therefore, we chose 30 pg/mL of HIV-1 p24 to test for biotin interference (Table 1). Interference by biotin was observed in both serum and plasma containing 30 pg/mL of p24 spiked with biotin at 200 and 400 ng/mL.

**Table 1.** Biotin Interference in Plasma and Serum Using Rapid Alere Determine HIV Combo Assay

	HIV 1 p24, pg/mL	Biotin, ng/mL	Plasma	Serum	Biotin interference
1	30	0	Positive	Positive	No
2	30	12.5	Positive	Positive	No
3	30	25	Positive	Positive	No
4	30	50	Positive	Positive	No
5	30	100	Positive	Positive	No
6	30	200	Negative	Negative	Yes
7	30	400	Negative	Negative	Yes
15	0	0	Negative	Negative	NA

There was no interference by biotin observed with serum or plasma containing 30 pg/mL HIV-1 p24 when biotin was spiked at concentrations ≤100 ng/mL.

For testing clinical samples, we selected six members from three different Seracare seroconversion panels that consisted of antigen-only positive samples, antibody-only positive samples, and samples that were both antigen positive and antibody positive as shown in Table 2. Biotin interference was observed in all categories of samples as given in Table 3. Biotin interference in the antigen portion of the assay is due to the competition between free biotin and biotinylated antibodies to bind to streptavidin. The variation in the biotin interference in different members of the same seroconversion panel is due to antigen and antibody dynamics. Antigenemia and antigen–antibody complexing occur as antibody production increases during the course of infection, reducing the detectable levels of HIV p24 concentration.^15^ This could contribute to biotin interference when detectable HIV p24 is lower as in panel members obtained in the later stage of seroconversion.

**Table 3.** Biotin Interference in Seroconversion Plasma Using Rapid Alere Determine HIV Combo Assay

ID	SC panel	Biotin, ng/mL	Antibody	Antigen
1	PBR975-05	100	NA	++ Faint
2	PBR975-05	200	NA	Interference
3	PBR975-05	400	NA	Interference
4	PBR946-03	100	NA	++ Faint
5	PBR946-03	200	NA	++ Faint
6	PBR946-03	400	NA	Interference
7	PBR946-04	100	NA	++
8	PBR946-04	200	NA	++
9	PBR946-04	400	NA	++
10	PBR947-02	100	++ Very Faint	++ Faint
11	PBR947-02	200	Interference	++ Faint
12	PBR947-02	400	Interference	Interference
13	PBR947-03	100	++ Faint	NA
14	PBR947-03	200	++ Faint	NA
15	PBR947-03	400	++	NA
16	PBR947-04	100	++	NA
17	PBR947-04	200	++	NA
18	PBR947-04	400	++	NA

Biotin interference in the antibody portion of the assay may be due to nonspecific binding and needs to be further investigated to determine the cause of interference. Biotin, like any other compound, is known to bind to other proteins and surfaces nonspecifically increasing background signal in many assays producing false positive results. Biotin interference in detection of anti-HIV-1 antibody in the seroconversion panel members in our study is due to the low concentration of gp41 antibodies. We have observed (unpublished data) that gp41 antibody concentrations are low in early infection and gradually increase after infection. Therefore, biotin interference at 200 and 400 ng/mL is detected with the early seroconversion panel member PBR947-02 and no biotin interference is detected with the seroconversion panel members PBR947-03 and PBR947-04.

This study was performed to assess the extent of biotin interference on antigen–antibody combo assays, and especially on the antigen portion of the assay. As HIV-1 p24 is an early marker for acute infection, it is important to understand the presence and scope of biotin interference, if any, in early diagnosis of HIV infection using a test whose design makes it susceptible to interference from biotin in samples.

Our study demonstrates that elevated levels of biotin interfere with performance of HIV-1 Ag/Ab combo POC immunoassay. Studies have shown that people who ingest >1 mg/day biotin may have serum concentrations of >8.6 ng/mL of biotin, levels that have been reported to interfere with other laboratory tests.^5^ Commercially available supplements may contain up to 100 mg of biotin, making high serum concentrations possible from consumer use of the supplements. The FDA has issued two public health safety communications addressing this concern.^9^ Therefore, it is important to consider biotin supplements as a potential source of false negative test results, especially in cases wherein the clinical signs and risk profile would place the individual at high risk for the condition being tested and the use of biotin supplements cannot be ruled out. Furthermore, it is important to consider biotin supplements as potential sources of falsely increased or decreased test results when selecting and interpreting laboratory test results.

## Abbreviations Used

PBS: phosphate-buffered saline
POC: point of care
